# History of infertility and pregnancy outcomes in Project Viva: a prospective study

**DOI:** 10.1186/s12884-022-04885-8

**Published:** 2022-07-07

**Authors:** Diana C. Soria-Contreras, Wei Perng, Sheryl L. Rifas-Shiman, Marie-France Hivert, Emily Oken, Jorge E. Chavarro

**Affiliations:** 1 Center for Nutrition and Health Research, National Institute of Public Health, Avenida Universidad No. 655, Santa Maria Ahuacatitlan, 62100 Cuernavaca, Morelos Mexico; 2 Present affiliation: Department of Nutrition, Harvard T.H. Chan School of Public Health, 655 Huntington Ave, Boston, MA 02115 USA; 3 Department of Epidemiology, Colorado School of Public Health, University of Colorado Denver, Anschutz Medical Campus, 13001 E. 17th Place, Aurora, CO 80045 USA; 4 Lifecourse Epidemiology of Adiposity and Diabetes (LEAD) Center, Colorado School of Public Health, University of Colorado Denver, Anschutz Medical Campus, 12474 East 19th Ave, Aurora, CO 80045 USA; 5 Division of Chronic Disease Research Across the Lifecourse, Department of Population Medicine, Harvard Medical School, and Harvard Pilgrim Health Care Institute, Landmark Center, 401 Park Drive, Suite 401 East, Boston, MA 02215 USA; 6 Diabetes Unit, Massachusetts General Hospital, 50 Staniford Street, Boston, MA 02114 USA; 7 Channing Division of Network Medicine, Department of Medicine, Brigham and Women’s Hospital and Harvard Medical School, 75 Francis St, Boston, MA 02115 USA

**Keywords:** Infertility, pregnancy outcome, systolic blood pressure, medically assisted reproduction, gonadotropins, GnRH agonists

## Abstract

**Background:**

Infertility has been associated with the risk of adverse pregnancy outcomes. It is not clear whether infertility and underlying causes of infertility or the use of medically assisted reproduction (MAR) therapies are responsible for the observed associations. In this study, we aimed to evaluate the association of history of infertility with pregnancy outcomes and identify whether the associations, if present, differed by subgroups defined by the use of MAR.

**Methods:**

Prospective study of 2201 pregnant women from the Boston-area Project Viva cohort. The exposure was history of infertility based on self-reported time to pregnancy ≥12 mo (or ≥ 6 mo if ≥35 y) or use of MAR; a diagnosis of infertility or claims for infertility treatments from medical records. The outcomes included: gestational glucose tolerance (gestational diabetes, impaired glucose tolerance, isolated hyperglycemia vs. normoglycemia), hypertensive disorders (gestational hypertension/preeclampsia vs. normotension), gestational weight gain (inadequate/excessive vs. adequate), systolic (SBP) and diastolic blood pressure, birthweight-for-gestational age z-score (tertile 2 and 3 vs. 1), preterm birth (<37 vs. ≥37 weeks at delivery), and birth outcome (pregnancy loss vs. live birth). We performed linear and logistic/multinomial regression analyses adjusted for age, race/ethnicity, age at menarche, pre-pregnancy BMI, and prenatal smoking.

**Results:**

Mean (SD) age was 32.0 (5.0) years, and 18.8% of women had history of infertility, 32.6% of whom used MAR. SBP across pregnancy was 0.72 mmHg higher in women with vs. without infertility (95% CI 0.02, 1.42). The associations were stronger among women who used MAR (β 1.32 mmHg, 95% CI 0.21, 2.44), especially among those who used gonadotropins or gonadotropin-releasing hormone [GnRH] agonists (β 1.91 mmHg, 95% CI 0.48, 3.35). Other outcomes were not associated with history of infertility.

**Conclusions:**

A history of infertility was associated with higher SBP during pregnancy, with stronger associations among those who used gonadotropins or GnRH agonists. Future studies are needed to confirm these findings and determine their clinical implications.

**Supplementary Information:**

The online version contains supplementary material available at 10.1186/s12884-022-04885-8.

## Background

Infertility is defined by the failure to achieve a clinical pregnancy after ≥12 months of regular, unprotected attempts at conception and affects 15.5% of couples in the United States (US) [1, 2]. Infertility encompasses a spectrum of reproductive dysfunction that includes secondary infertility (e.g., infertility after a previously established pregnancy), couples who meet the clinical definition of infertility but ultimately become pregnant without medical assistance, and those who require medical treatments to achieve a pregnancy [3]. Infertility has implications for health status beyond the reproductive consequences, including an increased risk of adverse pregnancy outcomes [4]. Improving our understanding of the role of infertility and pregnancy outcomes has been identified as a research priority in the US [5].

There is consistent evidence regarding the relationship of infertility with hypertensive disorders of pregnancy (HDP) and preterm birth [4, 6–10]. Some evidence also suggests that infertility is associated with a greater risk of gestational diabetes mellitus (GDM) and small birth size. However, these findings are less consistent, with some studies showing positive associations [9, 11–13] and others showing no associations [6, 8]. Two mechanisms have been proposed to explain the adverse associations between infertility and pregnancy outcomes, one related to underlying causes of infertility and another that revolves around the use of medically assisted reproduction (MAR) [14]. While the former suggests that adverse consequences associated with infertility are due to pre-existing pathophysiology, the latter indicates an additional risk to the gravida due to MAR. Clarifying which mechanism is at play has important implications for clinical care and intervention strategies.

In this study, we evaluated the association between history of infertility and pregnancy outcomes using prospectively collected information from the birth cohort Project Viva. As a secondary aim, we identified whether the associations, if present, differed by subgroups defined by the use of MAR.

## Methods

### Study population

This secondary analysis includes women participating in Project Viva, an ongoing prospective cohort of mother-child pairs recruited between 1999 and 2002 from Atrius Harvard Vanguard Medical Associates at around ten weeks gestation. Details on recruitment and eligibility are described elsewhere [15]. Our sample included 2276 women with singleton pregnancies who had a live newborn (*n* = 2100) or a pregnancy loss (*n* = 176). For 30 women who participated with two different pregnancies, we only considered the first pregnancy enrolled in the study. We further excluded women younger than 18 years at enrollment (*n* = 30) and those with preexistent chronic hypertension, type 1, or type 2 diabetes (*n* = 45). Our final analytical sample included 2201 women with data on the exposure and at least one pregnancy outcome. All participants provided written informed consent at enrollment. The institutional review board of Harvard Pilgrim Health Care approved all study protocols in line with ethical standards established by the Declaration of Helsinki.

### Exposure: infertility for the index pregnancy

Our primary exposure was history of infertility for the index pregnancy (yes/no), assessed via three sources of information. First, we used questionnaire data from the first study visit inquiring on whether they were actively trying to become pregnant and, if so, the number of cycles it took them to become pregnant. We classified women reporting ≥12 cycles to become pregnant, or ≥ 6 cycles if ≥35 years of age, as infertile [2]. Second, we used information obtained from the women’s medical records on history of infertility – specifically, whether they had a diagnosis of infertility (International Classification of Diseases-9 code 628.9 entries before the last menstrual period [LMP] + 60 days), a claim for infertility consultation or services, or prescriptions for fertility medications (e.g., clomiphene citrate, gonadotropins, or gonadotropin-releasing hormone agonists before LMP + 14 days). Lastly, women completed a detailed reproductive history on a follow-up questionnaire administered ~18 years after delivery. In this questionnaire, participants were asked if it had taken them ≥12 months to become pregnant, or ≥ 6 cycles if ≥35 years of age, or if they had used medical treatment for this purpose in each of their past pregnancies. If they responded “yes” to either question for the index pregnancy, we classified them as having history of infertility. If the time to pregnancy reported in this questionnaire was different from the information reported at the first study visit, we only considered the latter.

To identify whether the associations differed between subgroups defined by the use of MAR, we further classified women with infertility as those who used vs. did not use MAR. The former category included women who had prescriptions for fertility medications abstracted from the medical records (e.g., clomiphene citrate, gonadotropins, or gonadotropin-releasing hormone agonists), or who, at the 18-year study visit, reported the use fertility medications or treatments for the index pregnancy (e.g., medications to induce ovulation such as clomiphene, gonadotropic injections, intrauterine insemination, or assisted reproductive technology [ART] including in-vitro fertilization [IVF], intracytoplasmic sperm injection, sperm donation, egg or embryo donation, other). The latter category included women who did not report any treatment to conceive and did not have prescriptions for fertility medications in the medical records.

### Outcomes: pregnancy outcomes

We were interested in several maternal and newborn outcomes. Women underwent a clinical glycemic screening at 26–28 weeks of gestation with a non-fasting glucose challenge test (GCT) [16]. The GCT consisted of the administration of a 50-g oral glucose load with venous blood sampled 1 h after the load. If the blood glucose was >140 mg/dL, the clinician referred the woman for a fasting 3-h, 100-g oral glucose tolerance test (OGTT). Abnormal OGTT results were a blood glucose >95 mg/dL at baseline, >180 mg/dL at 1 h, >155 mg/dL at 2 h, or > 140 mg/dL at 3 h. We classified women into the following categories: normoglycemic, isolated hyperglycemia (e.g., an abnormal GCT but a normal OGTT), impaired glucose tolerance (IGT) (e.g., one abnormal value on the OGTT), and GDM (e.g., at least two abnormal values on the OGTT).

We identified women with gestational hypertension (GH) or preeclampsia from the outpatient and hospital medical records. GH included women who did not have chronic hypertension but developed elevated systolic blood pressure (SBP, >140 mmHg) or diastolic blood pressure (DBP, >90 mmHg) on ≥2 occasions after 20 weeks gestation. We classified women as having preeclampsia if they did not have chronic hypertension but developed high SBP (>140 mmHg) or DBP (>90 mmHg) in addition to proteinuria or if they had chronic hypertension and developed proteinuria after 20 weeks gestation [17]. We combined GH and preeclampsia for analysis. We extracted clinical measures of blood pressure from the medical records and calculated average SBP and DBP within each trimester of pregnancy. We defined the first trimester as LMP to 91 days, the second trimester as 92 to 182 days, and the third trimester as 183 days to delivery. For analyses of average blood pressure, we present results for both SBP and DBP but focus the interpretation of results on SBP because it is a stronger predictor of long-term cardiovascular outcomes than DBP [18–20].

Using clinical prenatal weights, we calculated total gestational weight gain (GWG) as the difference between the last pregnancy weight, measured <4 weeks from delivery, and self-reported pre-pregnancy weight. Then, we categorized GWG as inadequate, adequate, or excessive based on the Institute of Medicine guidelines [21].

We obtained information on the newborn’s sex, birthweight, and delivery date from the medical delivery records. We calculated birthweight-for-gestational age and sex z-scores (BWZ) based on US national reference data [22]. We categorized it in tertiles due to the relatively small sizes in some cells when using conventional categories of birth size (small-, appropriate-, and large-for-gestational-age). We calculated gestational age at birth in weeks by subtracting the date of the LMP from the date of delivery; we used the 2nd-trimester ultrasound in cases where the estimated delivery date by LMP differed by >10 days [17]. If infants were born <37 weeks of gestation, we classified them as preterm. We categorized birth outcome as a live birth or pregnancy loss (stillbirth or miscarriage) based on information from the outpatient and hospital medical records and study disenrollment data.

### Covariates

At enrollment, women reported their age, race/ethnicity, education level, marital status, annual household income, parity, and prenatal smoking habits via a self-administered questionnaire. We calculated pre-pregnancy body mass index (BMI, kg/m^2^) from self-reported pre-pregnancy weight and height. At a study visit conducted ~13 years after enrollment, the participants provided information on age at their first menstrual period.

### Statistical analysis

Prior to the formal analysis, we assessed the distribution of maternal characteristics across categories of infertility and compared them using mean (standard deviation [SD]) for numerical variables or frequencies and proportions for categorical variables.

In our primary analysis, we examined the associations between infertility (yes vs. no [reference]) and pregnancy outcomes using logistic regression models for HDP, preterm birth and birth outcome, multinomial logistic regression for gestational glucose tolerance status, GWG, tertiles of BWZ, and linear mixed regression models for SBP and DBP across the three trimesters of pregnancy. We selected potential confounders and precision covariates for model adjustment based on *a priori* knowledge and a literature review. The variables under study are depicted in a Directed Acyclic Graph included as a supplemental figure (Fig. S1). The final inclusion of covariates in the models was based on bivariate associations with the exposure/outcomes, and their impact on the effect estimates. For each of the outcomes, we constructed a series of multivariable models. Model 1 included age at enrollment (18–29, 30–34, ≥35 years), race/ethnicity (white, Black, Asian, Hispanic, other), and age at menarche (<12, 12–14, ≥15 years). Model 2 was additionally adjusted for pre-pregnancy BMI (continuous) and prenatal smoking habits (former smoker, smoker during pregnancy, never smoker). We evaluated the presence of an interaction between age at enrollment and infertility using an interaction term, but this was not significant for any of the outcomes (*p* > 0.05); therefore, we did not include the interaction term. For all the outcomes, additional adjustment for education, marital status, household income, family history of type 2 diabetes (for glucose tolerance status), and family history of hypertension (for HDP/blood pressure) did not influence the results substantially; therefore, we did not include these variables in the final models.

For SBP and DBP, we used linear mixed regression models to account for the repeated blood pressure measurements across pregnancy. We had up to 3 repeated measurements per woman (e.g., trimester-specific averages). These models included a random intercept and slope, with unstructured covariance to account for within-woman correlations. The interaction between infertility and the trimester of pregnancy at blood pressure assessment was not significant; hence, we did not include it in the final model. The estimates from these models may be interpreted as the mean difference in SBP and DBP across pregnancy with respect to baseline infertility status.

In a secondary analysis, we classified women as having history of infertility with and without MAR vs. those without infertility as the referent. We evaluated associations with those outcomes for which we detected significant associations in the primary analysis. We considered models adjusted for the same covariates previously described. In our last analysis, we subclassified women with MAR by the type of medication reported, which in most cases were medications to induce ovulation. We subclassified medications into three groups: 1) clomiphene citrate (CC) (alone or with gonadotropins or gonadotropin-releasing hormone [GnRH] agonists, 2) gonadotropins or GnRH agonists without clomiphene, 3) other (unspecified medications to induce ovulation, other treatment or no treatment specified). For this analysis, we compared the associations of each of the three groups vs. two different referent groups: women without history of infertility and women with history of infertility without MAR, to parse out the specific associations of each medication from the global association of infertility.

We conducted two sensitivity analyses. First, we excluded women classified as having history of infertility (*n* = 2) (primary analysis) and history of infertility with MAR (*n* = 10) (subgroup analyses) based solely on information reported ~18 years after delivery, hence more likely subject to recall bias and misclassification. The results were identical to those of the main analyses; therefore, only these are presented. Second, we excluded 39 women diagnosed with polycystic ovary syndrome (PCOS) before the index pregnancy to assess whether this condition could explain our main findings (diagnosis abstracted from medical records or self-reported at the 18-year study visit).

To deal with missing covariate data, we conducted chained equation multiple imputation to generate 50 imputed data sets using an imputation model that included the exposure, outcomes, and covariates under study. Missingness varied from <2% for race/ethnicity and pre-pregnancy BMI to 52% for age at menarche. The imputed data sets were combined and analyzed using MI ESTIMATE in Stata 16.

We conducted all the analyses in Stata 16 (StataCorp L.P., College Station, Texas).

## Results

In this study, 18.8% (*n* = 414) of the participants had history of infertility for the index pregnancy, and 32.6% (*n* = 135) of women with infertility used MAR (Table 1). The distribution by type of treatment in women with MAR was as follows: 34.1% (*n* = 46) used CC, 58.5% (*n* = 79) used gonadotropins or GnRH agonists, and 7.4% (*n* = 10) reported other treatments. Around 50% of women with infertility were ≥ 35 years at enrollment, while only 24% of women without infertility were in this age category. Almost 75% of women with infertility were non-Hispanic white and college-educated. The proportion of women with these characteristics among those without infertility was around 65%. In bivariate analysis, women with infertility had a significantly higher SBP and DBP during the first and second trimesters of pregnancy vs. women without infertility (Table S1).

**Table 1:** Participants’ characteristics by history of infertility for the index pregnancy (*n* = 2201)^a^

	History of infertility			
	Yes ***n*** = 414 (18.8%)^**b**^		No ***n*** = 1787 (81.2%)	
Maternal characteristics at enrollment				
	**Mean**	**SD**	**Mean**	**SD**
*Pre-pregnancy BMI, kg/m*^*2*^	25.1	5.6	24.8	5.6
	**N**	**%**	**N**	**%**
*Age*				
18–29 years	63	15.2	602	33.7
30–34 years	151	36.5	764	42.8
≥ 35 years	200	48.3	421	23.6
*Race/ethnicity*				
White	304	74.3	1149	65.3
Asian	19	4.6	104	5.9
Black	52	12.7	306	17.4
Hispanic	19	4.6	135	7.7
Other	15	3.7	66	3.8
*College graduate*				
No	110	26.9	661	37.6
Yes	299	73.1	1099	62.4
*Married/cohabiting*				
No	12	2.9	165	9.4
Yes	397	97.1	1594	90.6
*Household income > $70,000/year*				
No	123	33.2	617	40.4
Yes	248	66.8	909	59.6
*Pregnancy smoking status*				
Former smoker	84	20.9	312	18.3
Smoked during pregnancy	32	8.0	233	13.6
Never smoker	286	71.1	1162	68.1
*Nulliparous*				
No	184	44.4	969	54.2
Yes	230	55.6	818	45.8
*Age at first period*				
< 12 years	37	17.7	144	16.8
12 to 14 years	153	73.2	615	71.8
≥ 15 years	19	9.1	97	11.3

*BMI* body mass index

^a^The sample includes 2201 women with data on the exposure and ≥ 1 pregnancy outcome. The description was conducted using non-imputed data. The sample may not add to 2201 due to missing covariate values

^b^Among women with infertility for the index pregnancy, 135 (32.6%) used medically assisted reproduction: 46 (34.1%) used clomiphene citrate alone, or with gonadotropins or gonadotropin-releasing hormone agonists, 79 (58.5%) reported gonadotropins or gonadotropin-releasing hormone agonists, and 10 (7.4%) used other treatments

In the unadjusted model (Table 2), women with history of infertility for the index pregnancy had higher odds of IGT vs. normoglycemia than those with no history of infertility (OR 1.83, 95% CI 1.04, 3.21); however, after adjustment for age, race/ethnicity, and age at menarche, the associations were slightly attenuated and included the null (OR 1.70, 95% CI 0.94, 3.06). Additional adjustment for pre-pregnancy BMI and prenatal smoking habits in model 2 yielded similar results. Mean SBP across pregnancy was higher in women with infertility than those without infertility, and the results were consistent across unadjusted and adjusted models (β 0.72 mmHg, 95% CI 0.02, 1.42) (Table 2, model 2). We noted similar associations for DBP, though the effect estimates were smaller in magnitude – approximately 2/3 that of SBP in the unadjusted model and model 1 – and became attenuated after fully accounting for covariates in model 2 (β 0.40 mmHg, 95% CI -0.12, 0.93). We did not observe consistent associations with the other pregnancy outcomes.

**Table 2:** Unadjusted and adjusted odds ratios (OR) or β coefficients for pregnancy outcomes in women with vs. without history of infertility^a^

	Unadjusted		Model 1		Model 2	
	**OR**	**95% CI**	**OR**	**95% CI**	**OR**	**95% CI**
Gestational glucose tolerance status (*n* = 1984)						
Isolated hyperglycemia vs. Normoglycemic	1.17	(0.79, 1.72)	0.99	(0.67, 1.48)	0.98	(0.65, 1.46)
Impaired glucose tolerance vs. Normoglycemic	1.83	(1.04, 3.21)	1.70	(0.94, 3.06)	1.67	(0.93, 3.02)
Gestational diabetes mellitus vs. Normoglycemic	1.02	(0.61, 1.68)	1.04	(0.62, 1.75)	1.02	(0.60, 1.72)
Hypertensive disorders of pregnancy (*n* = 1988)						
Gestational hypertension/preeclampsia vs. Normotensive	0.87	(0.59, 1.28)	0.91	(0.61, 1.35)	0.88	(0.59, 1.31)
Gestational weight gain (*n* = 1971)						
Inadequate vs. Adequate	1.17	(0.80, 1.71)	1.19	(0.80, 1.76)	1.16	(0.78, 1.72)
Excessive vs. Adequate	1.18	(0.91, 1.54)	1.24	(0.94, 1.62)	1.22	(0.93, 1.61)
Birthweight-for-gestational age and sex z-scores (*n* = 2028)^b^						
Tertile 1 vs. Tertile 2	0.89	(0.68, 1.17)	1.01	(0.76, 1.34)	1.01	(0.76, 1.35)
Tertile 3 vs. Tertile 2	0.97	(0.74, 1.28)	0.94	(0.71, 1.25)	0.92	(0.69, 1.22)
Preterm birth (n = 2029)						
Yes vs. No	1.24	(0.82, 1.87)	1.41	(0.92, 2.16)	1.40	(0.91, 2.13)
Birth outcome (*n* = 2201)						
Pregnancy loss vs. Live birth ^c^	1.20	(0.82, 1.76)	1.06	(0.72, 1.58)	1.09	(0.73, 1.62)
	**β**	**95% CI**	**β**	**95% CI**	**β**	**95% CI**
Average SBP across pregnancy, mmHg^d^	0.89	(0.13, 1.64)	0.93	(0.17, 1.69)	0.72	(0.02, 1.42)
Average DBP across pregnancy, mmHg^d^	0.59	(0.05, 1.13)	0.57	(0.01, 1.13)	0.40	(−0.12, 0.93)

Model 1: age at enrollment (18–29, 30–34, ≥35 years), race/ethnicity (white, Black, Asian, Hispanic, other), age at menarche (<12, 12–14, 15 years)

Model 2: model 1 + pre-pregnancy BMI (continuous) and pregnancy smoking status (former, smoker during pregnancy, never smoker)

*DBP* diastolic blood pressure, *SBP* systolic blood pressure

^a^The sample may not add 2201 in some outcomes due to missing outcome data. Women without history of infertility are the reference group

^b^Mean (SD) birthweight-for-gestational age and sex z-scores in tertile 1: −0.86 (0.48); tertile 2, 0.17 (0.24); tertile 3, 1.25 (0.50)

^c^Pregnancy loss includes stillbirth (*n* = 9) and miscarriage (*n* = 163)

^d^β coefficient obtained from mixed regression models; based on *n* = 2023

We then assessed differences in SBP across pregnancy with respect to infertility history after further grouping infertile women based on the use of MAR. We found that women who had infertility and used MAR had ~1.3 mmHg higher SBP across pregnancy than those without infertility in unadjusted and adjusted models (Table 3). The estimates for infertility without MAR were in the same direction but weaker in magnitude and included the null (β 0.42 mmHg, 95% CI -0.41, 1.25).

**Table 3:** Unadjusted and adjusted β coefficients for systolic blood pressure in women with vs. without infertility, according to the use of MAR^a^

	Unadjusted		Model 1		Model 2	
Average SBP across pregnancy, mmHg^b^	β	95% CI	β	95% CI	β	95% CI
Without history of infertility (*n* = 1646) [ref]	0.00		0.00		0.00	
History of infertility without MAR (*n* = 250)	0.68	(−0.22, 1.57)	0.66	(−0.24, 1.56)	0.42	(−0.41, 1.25)
History of infertility with MAR (*n* = 127)	1.30	(0.09, 2.52)	1.46	(0.25, 2.67)	1.32	(0.21, 2.44)

Model 1: age at enrollment (18–29, 30–34, ≥35 years), race/ethnicity (white, Black, Asian, Hispanic, other), age at menarche (<12, 12–14, 15 years)

Model 2: model 1 + pre-pregnancy BMI (continuous) and pregnancy smoking status (former, smoker during pregnancy, never smoker)

*MAR* medically assisted reproduction, *SBP* systolic blood pressure

^a^The sample may not add 2201 due to missing outcome data

^b^β coefficient obtained from mixed regression models

In the last analysis, we subclassified women with MAR by the type of therapy reported, which mainly consisted of medications to induce ovulation. Compared to women without infertility, those with infertility who reported gonadotropins or GnRH agonists had a 1.9 mmHg higher SBP across pregnancy (95% CI 0.48, 3.35). When we considered women with infertility without MAR as the reference, SBP across pregnancy remained higher among GnRH agonist users, although the estimate was slightly weaker in magnitude and included the null (β 1.49 mmHg, 95% CI -0.09, 3.08) (Table 4, model 2). We did not observe consistent differences in SBP among CC users or those who reported other/unspecified treatments, although our sample size was small in these subgroups. We observed similar results in a sensitivity analysis excluding 39 women with PCOS before the index pregnancy (Tables S2, S3 and S4).

**Table 4:** Unadjusted and adjusted β coefficients for systolic blood pressure in women with vs. without infertility, according to the use of MAR and type of medication^a^

Average SBP across pregnancy, mmHg^b^	Unadjusted		Model 1		Model 2	
	β	95% CI	β	95% CI	β	95% CI
*Reference: without history of infertility*						
Without history of infertility (*n* = 1646) [ref]	0.00		0.00		0.00	
History of infertility without MAR (*n* = 250)	0.68	(−0.22, 1.57)	0.66	(−0.24, 1.56)	0.42	(−0.41, 1.25)
History of infertility with CC (*n* = 43)^c^	0.86	(−1.17, 2.90)	1.03	(−0.99, 3.04)	0.80	(−1.07, 2.66)
History of infertility with gonadotropins or GnRH agonists (*n* = 74)^d^	1.40	(−0.17, 2.97)	1.68	(0.12, 3.23)	1.91	(0.48, 3.35)
History of infertility with other medications/treatments (*n* = 10)^e^	2.42	(−1.75, 6.60)	1.75	(−2.38, 5.87)	−0.78	(−4.59, 3.04)
*Reference: history of infertility without MAR*						
Without history of infertility (*n* = 1646)	−0.68	(−1.57, 0.22)	−0.66	(−1.56, 0.24)	−0.42	(−1.25, 0.41)
History of infertility without MAR (*n* = 250) [ref]	0.00		0.00		0.00	
History of infertility with CC (*n* = 43)^c^	0.19	(−1.99, 2.37)	0.37	(−1.78, 2.52)	0.38	(−1.61, 2.37)
History of infertility with gonadotropins or GnRH agonists (*n* = 74)^d^	0.73	(−1.02, 2.47)	1.02	(−0.71, 2.74)	1.49	(−0.09, 3.08)
History of infertility with other medications/treatments (*n* = 10)^e^	1.75	(−2.50, 5.99)	1.09	(−3.10, 5.28)	−1.19	(−5.07, 2.68)

Model 1: age at enrollment (18–29, 30–34, ≥35 years), race/ethnicity (white, Black, Asian, Hispanic, other), age at menarche (<12, 12–14, 15 years)

Model 2: model 1 + pre-pregnancy BMI (continuous) and pregnancy smoking status (former, smoker during pregnancy, never smoker)

*CC* clomiphene citrate, *GnRH* Gonadotropin-releasing hormone, *MAR* medically assisted reproduction, *SBP* systolic blood pressure

^a^The sample may not add 2201 due to missing outcome data

^b^β coefficient obtained from mixed regression models

^c^Includes CC alone (*n* = 16), or CC + gonadotropins or GnRH agonists (*n* = 27)

^d^Includes gonadotropins or GnRH agonists without CC

^e^Includes unspecified medications to induce ovulation, women who reported other treatments or did not specify treatment

## Discussion

### Main findings

In this racially and ethnically diverse prospective cohort of women, those who experienced infertility before pregnancy had 0.7 mmHg higher SBP across pregnancy, an association that was even more pronounced (~1.3 mmHg) for women who conceived using MAR. In our study, 93% of women with infertility and MAR reported the use of hormonal medications to induce ovulation, most of which were gonadotropins or GnRH agonists. Women who used these medications had even higher SBP across pregnancy (~1.9 mmHg) than women without infertility. However, they also exhibited a somewhat higher SBP than women with infertility without MAR (~1.5 mmHg). We noted similar findings for DBP, though the estimates were smaller in magnitude and became attenuated after adjustment for pre-pregnancy BMI and prenatal smoking. Although these findings need to be confirmed in other studies, they suggest that the use of gonadotropins or GnRH agonists may be risk factors for systolic hypertension.

We did not observe consistent associations of infertility with gestational glucose tolerance, HDP, BWZ, preterm birth, or birth outcome. However, it is worth mentioning that women with infertility had almost twice the odds of IGT in the unadjusted models, but the estimates became attenuated after accounting for covariates. We also observed strong effect estimates for preterm birth, but the confidence intervals included the null in all the models.

### Interpretation

There are some plausible biological mechanisms to support our findings concerning SBP. Medications to induce ovulation, such as gonadotropins and GnRH agonists, are used to achieve supraphysiological levels of estradiol [23]. Estrogens affect vascular health and blood pressure [24]. For example, their exogenous administration in oral contraceptives or hormone replacement therapy has shown to increase blood pressure through activation of the renin-angiotensin-aldosterone system (RAAS), increased activity of the calcium channels, among other mechanisms [24–26]. Therefore, the rise to supraphysiological levels of estrogens as part of fertility treatments may have a similar impact on the internal hormonal milieu and blood pressure. A recent study showed increased arterial stiffness and heart rate in women undergoing controlled ovarian hyperstimulation for IVF [27]. An analysis of eight women with infertility and ovarian stimulation showed increased activation of the RAAS [28], which, as was mentioned before, is a crucial regulator of blood pressure [24]. In these scenarios, MAR treatment, in particular, that with gonadotropins or GnRH agonists, may be imparting additional risk on top of any pre-existing effects of infertility on pregnancy outcomes. Alternatively, using these medications could be a marker of the severity of infertility. Under this scenario, women with a more severe form of infertility would seek MAR and experience the worst outcomes.

Regarding the lack of consistent associations with the adverse pregnancy outcomes, it is possible that the small number of participants with IGT or preterm birth led to the imprecise estimates we observed. Despite the consistent associations with SBP, we did not observe associations with HDP. Contrary to our findings, Luke et al. found a 12% higher risk of gestational hypertension among women with infertility [4]. The inconsistent results may be related to a lack of statistical power given our study’s relatively small number of women with HDP. Future, adequately powered studies are needed to corroborate these associations.

### Strengths and limitations

Strengths of this study include the prospective design and the use of different sources of information to identify women with history of infertility and MAR. Additional strengths are the ascertainment of pregnancy outcomes from medical records instead of self-report, which is subject to bias, the racially/ethnically diverse study sample, and rich covariate data. Also, the inclusion of parous women recruited from the general population, as opposed to women with specific conditions related to infertility recruited from clinical settings such as fertility clinics, thereby making our findings more generalizable to reproductive-aged women in the US.

This study also has some limitations. First, we cannot rule out the possibility of exposure misclassification due to recall bias or incomplete interview or medical record data. Recall bias is a particular concern when the time to pregnancy is reported years after delivery [29]. However, when we excluded cases of infertility that were identified solely with information reported ~18 years after delivery, the results were virtually the same. Second, we did not have enough participants with reported use of ART to conduct subgroup analyses based on these types of therapies. Furthermore, when the cohort was set up, the participants could get ART outside of Atrius Harvard Vanguard Medical Associates. Therefore, it is possible that some women who used gonadotropins or GnRH agonists underwent ART and were not captured as such in the medical records. Third, underlying causes of infertility might influence the choice of MAR and impact pregnancy outcomes. Therefore, an additional limitation is the lack of information on causes of infertility other than PCOS (e.g., endometriosis). Fourth, we did not have information to characterize other factors that have been implicated in the activation of the RAAS during pregnancy and that may have an impact on blood pressure. Some of these factors include the specific IVF protocol and the number of corpus lutea at conception [30]. Future studies should try to understand the mechanisms behind the complex associations between infertility, MAR, and blood pressure. Fifth, as mentioned before, we may have been underpowered to detect associations with some outcomes in the main analysis and with SBP in subgroup analyses. Finally, this was an associational analysis from which we cannot determine causality.

## Conclusions

In this study, we found that women with history of infertility before the index pregnancy, especially those who used gonadotropins or GnRH agonists, had a higher SBP across pregnancy. Future studies are needed to confirm these findings and determine their clinical implications for both mother and offspring.

## Supplementary Information

**Additional file 1: Table S1.** Distributions of pregnancy outcomes by history of infertility for the index pregnancy (*n* = 2201). **Table S2.** Unadjusted and adjusted β coefficients for systolic blood pressure in women with vs. without infertility, excluding 39 women with PCOS. **Table S3.** Unadjusted and adjusted β coefficients for systolic blood pressure in women with vs. without infertility, according to the use of MAR and excluding 39 women with PCOS. **Table S4.** Unadjusted and adjusted β coefficients for systolic blood pressure in women with vs. without infertility, according to the use of MAR and type of medication, excluding 39 women with PCOS. **Figure S1.** Conceptual Directed Acyclic Graph depicting the variables under study.

## Data Availability

The datasets generated and/or analyzed during the current study are not publicly available due to privacy/ethical restrictions but are available from the corresponding author on reasonable request.

## Abbreviations

ART: Assisted reproductive technology
BMI: Body mass index
BWZ: Birthweight-for-gestational age and sex z-scores
CC: Clomiphene citrate
DBP: Diastolic blood pressure
GCT: Glucose challenge test
GDM: Gestational diabetes mellitus
GH: Gestational hypertension
GnRH: Gonadotropin-releasing hormone
GWG: Gestational weight gain
HDP: Hypertensive disorders of pregnancy
IVF: In-vitro fertilization
LMP: Last menstrual period
MAR: Medically assisted reproduction
OGTT: Oral glucose tolerance test
PCOS: Polycystic ovary syndrome
SBP: Systolic blood pressure
SD: Standard deviation
US: United States
