# Supplementary material for: History of infertility and pregnancy outcomes in Project Viva: a prospective study
Source: BMC Pregnancy Childbirth. 2022 Jul 7;22:549. doi: 10.1186/s12884-022-04885-8 (PMC9261051; doi:10.1186/s12884-022-04885-8)
Supplement: Supplementary file 1 — Additional file 1: Table S1. Distributions of pregnancy outcomes by history of infertility for the index pregnancy (n = 2201). Table S2. Unadjusted and adjusted β coefficients for systolic blood pressure in women with vs. without infertility, excluding 39 women with PCOS. Table S3. Unadjusted and adjusted β coefficients for systolic blood pressure in women with vs. without infertility, according to the use of MAR and excluding 39 women with PCOS. Table S4. Unadjusted and adjusted β coefficients for systolic blood pressure in women with vs. without infertility, according to the use of MAR and type of medication, excluding 39 women with PCOS. Figure S1. Conceptual Directed Acyclic Graph depicting the variables under study. [file 12884_2022_4885_MOESM1_ESM.docx]

**History of infertility and pregnancy outcomes in Project Viva: a prospective study**

**Supplemental tables and figure**

| Table S1. Distributions of pregnancy outcomes by history of infertility for the index pregnancy (n=2201) ^a^ | | | | |
| --- | --- | --- | --- | --- |
|  | **History of infertility** | | | |
|  | **Yes n=414 (18.8%)** | | **No n=1787 (81.2%)** | |
| PREGNANCY OUTCOMES | **N** | **%** | **N** | **%** |
| *Gestational glucose tolerance status* |  |  |  |  |
| Normoglycemic | 300 | 80.2 | 1340 | 83.2 |
| Isolated hyperglycemia | 36 | 9.6 | 138 | 8.6 |
| Impaired glucose tolerance | 18 | 4.8 | 44 | 2.7 |
| Gestational diabetes mellitus | 20 | 5.3 | 88 | 5.5 |
| *Hypertensive disorders of pregnancy* |  |  |  |  |
| Normotensive | 335 | 90.8 | 1459 | 89.6 |
| Gestational hypertension | 22 | 6.0 | 111 | 6.8 |
| Preeclampsia | 12 | 3.3 | 59 | 3.6 |
| *Gestational weight gain* |  |  |  |  |
| Inadequate | 49 | 13.2 | 205 | 12.8 |
| Adequate | 97 | 26.2 | 473 | 29.5 |
| Excessive | 224 | 60.5 | 923 | 57.7 |
| *Birthweight-for-gestational age*  *and sex z-scores ^b^* |  |  |  |  |
| Tertile 1 | 121 | 32.1 | 568 | 34.4 |
| Tertile 2 | 129 | 34.2 | 538 | 32.6 |
| Tertile 3 | 127 | 33.7 | 545 | 33.0 |
| *Preterm birth (<37 weeks)* |  |  |  |  |
| No | 345 | 91.5 | 1537 | 93.0 |
| Yes | 32 | 8.5 | 115 | 7.0 |
| *Birth outcome* |  |  |  |  |
| Live birth | 377 | 91.1 | 1652 | 92.4 |
| Pregnancy loss ^c^ | 37 | 8.9 | 135 | 7.6 |
|  | **Mean** | **SD** | **Mean** | **SD** |
| Systolic blood pressure |  |  |  |  |
| 1st trimester, mmHg | 112.1 | 8.3 | 111.0 | 8.6 |
| 2nd trimester, mmHg | 111.0 | 7.7 | 110.1 | 7.5 |
| 3rd trimester, mmHg | 113.1 | 8.4 | 112.5 | 7.6 |
| Diastolic blood pressure |  |  |  |  |
| 1st trimester, mmHg | 70.0 | 6.1 | 69.1 | 6.5 |
| 2nd trimester, mmHg | 68.0 | 5.2 | 67.3 | 5.6 |
| 3rd trimester, mmHg | 70.1 | 5.5 | 69.9 | 5.8 |
| ^a^ The sample includes 2201 women with data on the exposure and ≥1 pregnancy outcome.  ^b^ Mean (SD) birthweight-for-gestational age and sex z-scores in tertile 1: -0.86 (0.48); tertile 2, 0.17 (0.24); tertile 3, 1.25 (0.50).  ^c^ Includes stillbirth (n=9) and miscarriage (n=163). | | | | |

| Table S2. Unadjusted and adjusted β coefficients for systolic blood pressure in women with vs. without infertility, excluding 39 women with PCOS ^a^ | | | | | | |
| --- | --- | --- | --- | --- | --- | --- |
|  | **Unadjusted** | | **Model 1** | | **Model 2** | |
|  | **β** | **95% CI** | **β** | **95% CI** | **β** | **95% CI** |
| Average SBP across pregnancy, mmHg ^b^ |  |  |  |  |  |  |
| Without history of infertility (n=1632) [ref] | 0.00 | | 0.00 | | 0.00 | |
| History of infertility (n=352) | 0.86 | (0.09, 1.63) | 0.91 | (0.13, 1.69) | 0.82 | (0.09, 1.54) |
| PCOS: polycystic ovary syndrome; SBP: systolic blood pressure.  ^a^ Excludes 39 women with PCOS before the index pregnancy.  ^d^ β coefficient obtained from mixed regression models.  Model 1: age at enrollment (18-29, 30-34, ≥35 years), race/ethnicity (white, Black, Asian, Hispanic, other), age at menarche (<12, 12-14, 15 years). Model 2: model 1 + pre-pregnancy BMI (continuous) and pregnancy smoking status (former, smoker during pregnancy, never smoker). | | | | | | |

| Table S3. Unadjusted and adjusted β coefficients for systolic blood pressure in women with vs. without infertility, according to the use of MAR and excluding 39 women with PCOS ^a^ | | | | | | |
| --- | --- | --- | --- | --- | --- | --- |
|  | **Unadjusted** | | **Model 1** | | **Model 2** | |
| Average SBP across pregnancy, mmHg ^b^ | **β** | **95% CI** | **β** | **95% CI** | **β** | **95% CI** |
| Without history of infertility (n=1632) [ref] | 0.00 | | 0.00 | | 0.00 | |
| History of infertility without MAR (n=245) | 0.63 | (-0.27, 1.53) | 0.62 | (-0.29, 1.53) | 0.46 | (-0.38, 1.30) |
| History of infertility with MAR (n=107) | 1.38 | (0.06, 2.69) | 1.58 | (0.27, 2.89) | 1.64 | (0.43, 2.85) |
| MAR: medically assisted reproduction; PCOS: polycystic ovary syndrome; SBP: systolic blood pressure.  ^a^ Excludes 39 women with PCOS before the index pregnancy.  ^b^ β coefficient obtained from mixed regression models.  Model 1: age at enrollment (18-29, 30-34, ≥35 years), race/ethnicity (white, Black, Asian, Hispanic, other), age at menarche (<12, 12-14, 15 years). Model 2: model 1 + pre-pregnancy BMI (continuous) and pregnancy smoking status (former, smoker during pregnancy, never smoker). | | | | | | |

| Table S4. Unadjusted and adjusted β coefficients for systolic blood pressure in women with vs. without infertility, according to the use of MAR and type of medication, excluding 39 women with PCOS ^a^ | | | | | | |
| --- | --- | --- | --- | --- | --- | --- |
| *Reference: without history of infertility* | | | | | | |
|  | **Unadjusted** | | **Model 1** | | **Model 2** | |
| Average SBP across pregnancy, mmHg ^b^ | **β** | **95% CI** | **β** | **95% CI** | **β** | **95% CI** |
| Without history of infertility (n=1632) [ref] | 0.00 | | 0.00 | | 0.00 | |
| History of infertility without MAR (n=245) | 0.63 | (-0.27, 1.54) | 0.62 | (-0.28, 1.53) | 0.46 | (-0.37, 1.30) |
| History of infertility with CC (n=41) ^c^ | 0.53 | (-1.55, 2.62) | 0.71 | (-1.35, 2.77) | 0.64 | (-1.27, 2.54) |
| History of infertility with gonadotropins or GnRH agonists (n=60) ^d^ | 1.98 | (0.25, 3.71) | 2.29 | (0.56, 4.01) | 2.60 | (1.01, 4.19) |
| History of infertility with other medications/treatments (n=6) ^e^ | 1.12 | (-4.25, 6.50) | 0.48 | (-4.83, 5.79) | -1.11 | (-6.01, 3.80) |
| *Reference: history of infertility without MAR* | | | | | | |
|  | **Unadjusted** | | **Model 1** | | **Model 2** | |
| Average SBP across pregnancy, mmHg ^b^ | **β** | **95% CI** | **β** | **95% CI** | **β** | **95% CI** |
| Without history of infertility (n=1632) | -0.63 | (-1.54, 0.27) | -0.62 | (-1.53, 0.28) | -0.46 | (-1.30, 0.37) |
| History of infertility without MAR (n=245) [ref] | 0.00 | | 0.00 | | 0.00 | |
| History of infertility with CC (n=41) ^c^ | -0.10 | (-2.32, 2.12) | 0.08 | (-2.11, 2.28) | 0.18 | (-1.85, 2.21) |
| History of infertility with gonadotropins or GnRH agonists (n=60) ^d^ | 1.35 | (-0.55, 3.25) | 1.66 | (-0.21, 3.54) | 2.14 | (0.41, 3.87) |
| History of infertility with other medications/treatments (n=6) ^e^ | 0.49 | (-4.94, 5.92) | -0.14 | (-5.49, 5.21) | -1.57 | (-6.51, 3.37) |
| CC: clomiphene citrate; GnRH: Gonadotropin-releasing hormone; MAR: medically assisted reproduction; PCOS: polycystic ovary syndrome; SBP: systolic blood pressure.  ^a^ Excludes 39 women with PCOS before the index pregnancy.  ^b^ β coefficient obtained from mixed regression models.  ^c^ Includes CC alone (n=14), or CC + gonadotropins or GnRH agonists (n=27)  ^d^ Includes gonadotropins or GnRH agonists without CC.  ^e^ Includes unspecified medications to induce ovulation, women who reported other treatments or did not specify treatment.  Model 1: age at enrollment (18-29, 30-34, ≥35 years), race/ethnicity (white, Black, Asian, Hispanic, other), age at menarche (<12, 12-14, 15 years). Model 2: model 1 + pre-pregnancy BMI (continuous) and pregnancy smoking status (former, smoker during pregnancy, never smoker). | | | | | | |

**Pregnancy outcomes** ^c^

**Infertility**

**Figure S1.** Conceptual Directed Acyclic Graph depicting the variables under study.

^a^ Age, race/ethnicity, age at menarche, pre-pregnancy BMI, prenatal smoking, education, marital status, and household income.

^b^ Family history of type 2 diabetes (for glucose tolerance status), and hypertension (for hypertensive disorders of pregnancy/blood pressure).

^c^ Hypertensive disorders of pregnancy, preterm birth, birth outcome, gestational glucose tolerance status, gestational weight gain, birthweight-for-gestational age and sex z-scores, systolic and diastolic blood pressure across pregnancy.

Potential confounders ^a^

Potential precision covariates ^b^
